# Anatomy of a nonhost disease resistance response of pea to *Fusarium solani*: PR gene elicitation via DNase, chitosan and chromatin alterations

**DOI:** 10.3389/fpls.2015.00373

**Published:** 2015-06-12

**Authors:** Lee A. Hadwiger

**Affiliations:** Department of Plant Pathology, Washington State UniversityPullman, WA, USA

**Keywords:** nonhost resistance, Pea endocarp system, Defense gene transcription, *Fusarium solani sp*., DNA conformation, Chromatin, Stalled RNA polymerase complexes, Pathogenesis-related (PR) genes

## Abstract

Of the multiplicity of plant pathogens in nature, only a few are virulent on a given plant species. Conversely, plants develop a rapid “nonhost” resistance response to the majority of the pathogens. The anatomy of the nonhost resistance of pea endocarp tissue against a pathogen of bean, *Fusarium solani* f.sp. *phaseoli* (Fsph) and the susceptibility of pea to *F. solani* f sp. *pisi* (Fspi) has been described cytologically, biochemically and molecular-biologically. Cytological changes have been followed by electron microscope and stain differentiation under white and UV light. The induction of changes in transcription, protein synthesis, expression of pathogenesis-related (PR) genes, and increases in metabolic pathways culminating in low molecular weight, antifungal compounds are described biochemically. Molecular changes initiated by fungal signals to host organelles, primarily to chromatin within host nuclei, are identified according to source of the signal and the mechanisms utilized in activating defense genes. The functions of some PR genes are defined. A hypothesis based on this data is developed to explain both why fungal growth is suppressed in nonhost resistance and why growth can continue in a susceptible reaction.

## Focused review of nonhost resistance in pea

### Nonhost resistance

The nonhost disease resistance response of plants is exceptionally stable, compared with the disease resistance from R genes typically manipulated with plant breeding techniques. This suggests in nature an individual plant species, such as pea, vigorously resists plant pathogenic organisms that are not found to have pea in their “host range.” Alternately the true pathogens of peas may also incite a response in pea tissue, but one that is inadequate to develop definitive resistance and eventually yields to susceptibility. During the evolution of pea pathogens some have acquired functional components such as toxins that enable them to successfully infect when the environmental conditions are right for infection. Alternately the loss of other components, such as elicitors (effectors), can allow the pathogen to avoid inciting a strong resistance response. A recent hypothesis of nonhost resistance viewed it as a multilayer defense with callose, lignin-like material and hydrogen peroxide in the cell wall in the first layer and an oxidative burst leading to cell death in the second layer (Zellerhoff et al., 2010).

The role of effectors in nonhost resistance in plants to filamentous plant pathogens covering a conventional view has been reviewed by Stam et al. (2014). The components suggested to contribute to the resistance response are manipulated by PAMPs (Pathogen-Associated Molecular Patterns) associating with PRRs (Pattern-Recognition Receptors) on the host cell surface that activate PTI (Pattern-Triggered Immunity). The genetic data of Lee et al. (2014), suggests that one or two dominant genes of pepper are involved in the recognition of RXLR effectors from Phytophthora infestans. PRRs are thought to occur in large families such as those for the Crinkler (CRN) family of the *Phytophtora* genus (Stam et al., 2013). Of a predicted 84 full length CRN genes ~30 had nuclear localization sequences and when expressed *in planta*, only a few CRN domains induced necrosis.

The current hypothesis and the data herein have been derived primarily from one legume system and describes a divergent view of effector/elicitor reception and recognition. Assay points have been centered within an early window of the defense of pea endocarp tissue. The vital players act within a “first layer” in which the total resistance response against a bean pathogen, Fsph, occurs within 6 h pi. The nonhost resistance develops following direct contact by the pathogen to this cuticle-less pea endocarp tissue without the requirement for cell wall penetration. Also the fungal spore growth is terminated well before significant cell death is detectable, thus the induction of nonhost resistance in pea endocarp tissue is assayed primarily via the mRNA from PR (pathogenesis-related) genes, cytological detection of suppressed fungal growth and molecular changes in pea chromatin. We propose that chromatin can function as a receptor structure for incoming elicitors or effectors.

## A hypothesis related to chromatin alteration; as a component of the anatomy of nonhost disease resistance in pea tissue

This anatomy view focuses on the interactions between pea tissue and two *Fusarium* pathogens, one a pea pathogen, F. solani f. sp. pisi (Fspi) and the other a bean pathogen *F.solani* f. sp. *phaseoli* (Fsph) (Hadwiger, 2008). The functional aspects of the interactions are described at the cytological, biochemical, and molecular levels in this report. There are currently multiple model systems (Zellerhoff et al., 2010; Rojas et al., 2012) utilized for looking at the signaling that initiates the nonhost resistance response, however, the pea-endocarp/*Fusarium*-macroconidial system was chosen for the following reasons.

## The pea tissue has a 6 h time window for resistance

The pea endocarp, the tissue exposed following the separation of the two halves of an excised immature pea pod, distinguishes between the pea pathogen (Fspi) and the bean pathogen (Fsph), (Hadwiger, 2008) as completely and reliably as when the inocula of the two pathogens are introduced to the stems of intact pea seedlings. In inoculated seedlings the symptoms of the defense develop over 20 days. The absence of a cuticle layer is a factor allowing the development of total resistance of endocarp tissue to Fsph inoculum to complete within 6 h. Additionally, the signaling and all of the combined processes for resistance development are synchronized across the entire exposed epidermal cells of this intact tissue, without the artifacts that are inherent to cell culture systems. Although all of the vital signaling has occurred within 6 h, the germinating spores have not penetrated the tissue and can be recovered, thus the progression of biochemical and molecular events can be monitored separately at any point previous to 6 h in both the host and pathogen.

### Defined responses

Many of the molecular processes involved in the interaction have been researched. The technologies for monitoring of the early host responses such as phytoalexin production, pathogenesis-related (PR) gene induction, enzymes of secondary pathways, chromatin changes, alteration in cytological features, etc. have been established (Hadwiger, 2008). Biochemically, it has been shown, that both an ongoing transcription and protein synthesis in plant tissue are required. More specifically, the transcription and translation of PR genes are necessary for resistance. One observation in support of this statement was demonstrated by employing what is known about the effect of heat shock on transcription and translation. Heat shock at 30°C for 1 h reprograms translation and initiates the inhibition of pre-mRNA splicing (Biamonti and Cacres, 2008). At the ribosome level there is a temporary absence of normal mRNAs soon replaced by newly transcribed mRNA disproportionally specific for heat shock proteins. A heat treatment of pea endocarp tissue negates the transcription/translation of PR proteins and there is a corresponding loss of resistance to Fsph (Hadwiger and Wagoner, 1983). Following a 9 h recovery period, PR proteins can again be translated and disease resistance returns. At the level of protein synthesis plant defenses are functionally related (Zellerhoff et al., 2010) and there is an overlap in these responses. Nonhost resistance to multiple “inappropriate” pathogens reportedly involves the robust regulation of overlapping, similar sets of PR genes. These enhanced gene responses involve more than PR genes and have long been observed in double-label experiments where the resistance-enhanced/control ratios appear within wide range of total plant proteins, observed in separations based on size and charge (von Broembsen and Hadwiger, 1972).

Similar, but more specific analyses of the enhanced gene transcription during the peak of the defense response, were analyzed by subjecting the accumulated mRNA to a “reticulocyte *in vitro* translation system” followed by two-dimensional separations of the products. The separation patterns again point to the similarities of the plant's disease response signaled by various pathogens or elicitors (Loschke et al., 1983). This result has been increasingly substantiated by screening for individual induced genes within complete genome libraries (Riggleman et al., 1985) with plus-minus hybridization techniques or the screening of large numbers selected genes in microarrays.

## The non-host disease response as an assay for eliciting compounds

The pea endocarp system has been used to assay abiotic enhancers and inhibitors to screen for components that can mimic or augment the responses elicited by pathogen or non-pathogen. Also an array of pharmacological compounds and physical treatments (e.g., UV light) capable of inducing defense responses were identified in the pea endocarp tissue assay (Hartney et al., 2007).

## Early release of biotic signals following fungal contact with the endocarp surface

There is an early release of hydrolytic enzymes from *Fusarium* spores following contact with the pea tissue. Enzymes such as cutinase (Woloshuk and Kollatukudy, 1986), and DNase (Klosterman et al., 2001) are released as the inoculum contacts pea tissue. Alternately, the pea tissue has constitutive levels of enzymes such as chitinase and β-glucanase (Mauch et al., 1984), each with N-terminal “SignalP” peptides that can enable their immediate transfer through membranes (Hadwiger, 2009). The contacting surfaces of the fungus wall and the plant cell wall change in the presence of these digesting enzymes followed by an accompanying presence of polymeric fragments. Those with established inducing capacity include pectic (Simons-Walker et al., 1983), and chitosan oligomers (Kendra et al., 1989). Chemically, chitosan is deacetylated chitin, thus fungal cell wall chitin is either deacetylated (Hadwiger and Line, 1981; El Gueddari et al., 2002) or the poly-glucosamine oligomers are released from the chitin molecule (which in its native form is 20% deacetylated) by chitinase (Hadwiger, 2013). The early release of FsphDNase is followed within 20 min by a uniform reduction in the density of nuclei in the pea surface cells (Hadwiger and Adams, 1978).

### Pea host responses

Within the first 2 h following inoculation with Fsph, pea DNA damage is detectable and the induction of pea PR genes commences (Hadwiger et al., 1995). Some of the early proteins translated have known functions that may relate to their potential to slow fungal growth and are described as follows:

1. *DRR206* codes for an enzyme associated with a secondary pathway toward (lignan) production (Seneviratne et al., 2014). This pea gene when transferred to canola has conferred resistance against a fungal pathogen of canola (Wang et al., 1999);
2. *DRR230* and *DRR39* code for pea defensins (Chiang and Hadwiger, 1991), with defined antimicrobial activity (Almeida et al., 2006);
3. *DRR49* (*PR-10*) codes for a product that enters the nucleus (Allaire and Hadwiger, 1994) and is a putative RNase. DRR49 trans-genetically confers resistance in potato to early blight (Chang et al., 1993).
4. Genes for secondary enzymes: The genes for some secondary pathway enzymes are induced such as phenylalanine ammonia-lyase (PAL), (Loschke et al., 1981) and chalcone synthetase (CHS), functioning in the production of chalcones. PAL and CHS are also intermediates in the production of lignan (Seneviratne et al., 2014), lignin, flavonoids and isoflavonoids, e.g., the phytoalexin, pisatin (Cruickshank and Perrin, 1962; DiCenzo and VanEtten, 2006).
5. The pea gene PR-1 is homologous with the PR1b gene in Arabidopsis. In Arabidopsis this gene has a PR-1 function and is a “non-expressor” of NPR1 which reportedly is a master, positive regulator of plant immunity (Yu et al., 2001).

## Characterization of response proteins

Although individually these genes when transferred to other plants behind a suitable promoter can add increments of resistance to authentic fungal and bacterial pathogens, a combination of these induced defense genes appear to be involved in complete resistance. A broader view of the proteins coded for by the induced resistance response was possible by translating the accumulated RNAs in an *in vitro* protein synthesis assay (Wagoner et al., 1982) noted above. The representation of the labeled gene products that are in greater abundance in two dimensional separations can be seen as patterns. These pattern changes reflect the initiation of different physiological responses. Significantly the protein patterns induced in pea tissue were all closely similar among the pea responses to fungal inoculations, chitosan treatments, and applications of a DNA-intercalating agent, actinomycin D (AD). Additionally, these patterns were dramatically different from those found in heat-shock and heavy metal treated endocarp tissue. The biotic gene-inducing agents deemed important in the pea/Fusarium interactions were the chitosan oligomers (Kendra et al., 1989) and the FsphDNase (Klosterman et al., 2001) discussed above.

## DNA and chromatin targets

Additionally, the actual chemical components capable of inducing the pea defense responses were evaluated by subjecting the pea endocarp tissue to all available regulatory components and subsequently monitoring the defense response in terms of phytoalexin accumulations, PR gene inductions and enhancement or suppression of the resistance to the pathogens (Hartney et al., 2007). The overwhelming majority of the components with positive effects on the defense responses were those capable of altering DNA or chromatin. These included, DNA cleaving, DNA intercalating, (Schwochau and Hadwiger, 1968) base substituting (Sander and Hadwiger, 1979), thymidine dimerizing (Hadwiger and Schwochau, 1971), psoralen crosslinking (Parsons and Hadwiger, 1998), nuclear protein competitors of DNA attachment, or effectors that potentially altered phosphoration of nuclear proteins (Isaac et al., 2009).

Concurrent with these investigations were advances in detecting pea chromosomal regions (QTLs) that were associated with disease resistance genetically. Some of these chromosomal regions encumbered sites in which some PR genes had been mapped (Pilet-Nayel et al., 2002; Prioul-Gervais et al., 2007)

Phosphatase inhibitors, such as calyculin A, were among the components activating the pea defense responses that have not been directly associated with chromatin/DNA changes (Hartney et al., 2007). Further efforts to follow the phosphorylation of nuclear proteins, such as histones H2A/H2B and the transcription factor, HMG A, discovered that in addition to the phosphorylation changes, these proteins were also being removed from chromatin via ubiquitination (Klostermann et al., 2003; Isaac et al., 2009). Thus evidence was provided that may implicate alterations of both DNA and nuclear proteins in initiating transcription of defense genes. These direct changes at the point of gene transcription may be another route for enabling read-through of PR genes by stalled RNA polymerase complexes (Wu and Snyder, 2008).

**Figure 1:**
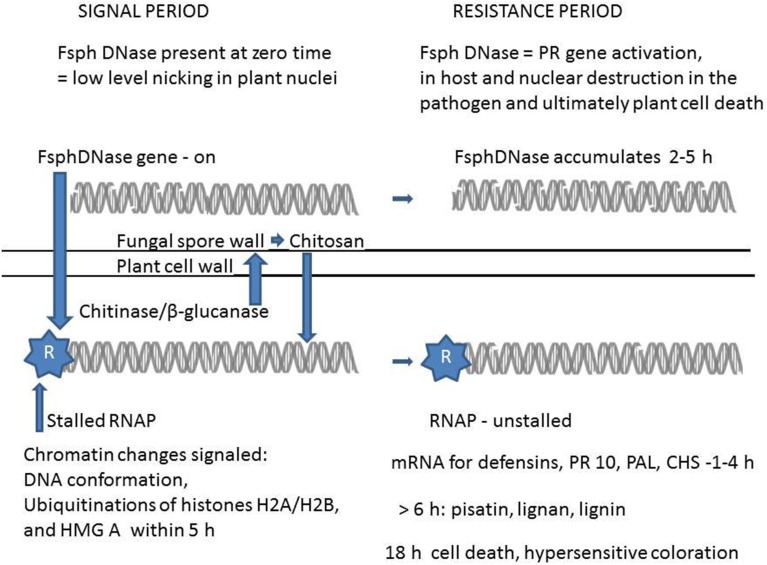
**A proposed scheme relating the role of DNase in altering plant chromatin and subsequently enhancing transcription and cell death**. The upper portion of the scheme relates the alterations of DNA within the fungal cell. At zero time the DNA helix is nearly intact in the germinating *Fusarium solani f. sp. phaseoli* (Fsph) spore, however FsphDNase is present and released as the spore germination begins. Following the entrance of FsphDNase into the plant cell (lower portion) there is a low level nicking of the DNA within the plant nucleus. The subsequent loosening of the DNA helical structure within the nucleosome assembly along with the ubiquitination of nuclear proteins, histone H2A/H2B and the architectural transcription factor (HMG A) frees the stalled RNA polymerase II complex (RNAP) enabling the defense genes to be transcribed. Of the pathogenesis-related (PR type) genes expressed are the defensins, (*DRR 39, DRR230*) with direct potent antifungal activity. *PR10* (*DRR49*) codes a putative RNase protein. Increases in phenylalanine ammonia lyase (*PAL*) and chalcone synthetase (*CHS*) are secondary plant enzymes that are essential for fungal suppressive components such as pisatin, lignan, and lignin. Since resistance to Fsph the inappropriate pathogen is complete in 6 h pi, the after 6 h appearance of these latter components and expression of cell death and hypersensitivity are not likely major contributors to disease resistance. The plant constitutively produces background levels of chitinase and β-glucanase. Induced levels appear starting 10 h pi. These secretable enzymes attack the regions of fungal wall chitin that is 80% N-acetyl-glucosamine and fungal wall β-glucan. Heptamers of chitosan oligomers are released that both induce PR genes and inhibit fungal growth. Within 2 h of contact with the plant tissue the nuclei within the growing tip of the germinating Fsph spore undergo DNA degradation. This disruption is adequate to prevent further fungal growth.

I propose that the chitosan and DNase elicitors of induced resistance described in Figure 1 are major players in the induced nonhost resistance response, however there are many other cell wall fragments, e.g. pectic oligomers and undescribed effectors present within the interchanges between fungal spores and plant cells. In other systems effectors, such as chitin oligomers, are proposed to complex with protein receptors (Wan et al., 2008) at the plant membrane level and through cascading transitions, eventually affect a transcription factor without directly acting on the DNA of chromatin. For example the phosphatase inhibitor, calyculin A potently induces pisatin production when applied to pea endocarp tissue at very low concentrations and at different concentrations can also increase or decrease the pea disease resistance response (Hartney et al., 2007). The mechanism(s) for calyculin A action remains unresolved. Another level of regulation is needed to explain the plant gene-for-fungal gene (Flor, 1971) specificity in peas that with the proper host R-gene, matched with the appropriate Avr gene of the pathogen, can dictation subsequent resistance or susceptibility outcomes. When nonhost and race specific resistance are viewed in terms of patterns of proteins *in vitro* transcribed mentioned above, there is a remarkable pattern similarity (Daniels et al., 1986) in the nonhost and race specific resistance responses. These observations in peas conform to the more recent observations of overlap in plant responses when the challenge is inflicted by different inappropriate pathogens (Zellerhoff et al., 2010).

## Background support for the proposed scheme of Figure 1

### Localization of elicitors

The FsphDNase gene has been cloned, its mRNA production monitored and the transfer of the protein product to the pea nucleus traced with specific anti-sera. Also the retention of its catalytic activity following localization in the plant nucleus was monitored with a DNase assay (Gerhold et al., 1993). The *in vivo* DNA degrading action has been documented in both host cells and fungal spores (Klosterman et al., 2001). Its individual function as a disease-resistance-inducing factor has been established transgenically in tobacco (Choi et al., 2004). The processing and action of fungal wall chitosan has also been documented (Kendra et al., 1989), its potential to localize in the DNA minor grove and subsequently cause DNA degradation have been reported (Hadwiger et al., 1989). Chitosan or chitosan oligomers can induce resistance in pea tissue against a true pea pathogen (Kendra et al., 1989) and directly suppress fungal growth by inhibiting both fungal DNA and RNA syntheses (Hadwiger et al., 1986).

### Chromosomal sensitivity

The mapping of chromosomal sites in peas for some general defense gene traits called Quantitative trait loci (QTLs) and the presence of some PR gene open-reading-frames located within (Pilet-Nayel et al., 2002) QTLs, have indicated that there are regions within pea chromosomes that are directly sensitive to insults to the chromatin structure. The early cytogeneticists, seeing the differential bulges of giant chromosomes of *Drosophila*, following applications of differential regulatory substances, were the first to realize the varying sensitivities within chromosomes (Bonner and Pardue, 1977) associated with enhanced transcription. Though there is currently no report describing “stalled” pea genes, this is a common phenomenon in other eukaryotic cells (Wu and Snyder, 2008). The promoters of these genes are often endowed with RNA polymerase II and the proper transcription factors but otherwise restricted until chromatin alterations make transcription possible.

### Chromatin modifications

As a general response to the pathogen challenge there is an ubiquitination of the nuclear proteins, histones H2A/H2B and HMG A. The removal of these functional proteins from sites containing PR gene sequences within 5 h pi has been documented using chromatin-immunoprecipitation (ChIP) techniques (Isaac et al., 2009).

### Cellular mechanism involved with chromatin alterations

There is a new realization that ubiquitination can be a component of transcription regulation and is not exclusively linked functionally to the previously designated role of simply degrading and recycling proteins. The proteins E2 and E 3 remain involved in H2B ubiquitination (Fuchs et al., 2015). Sequential ubiquitination and deubiquitination of histones as well as cooperation among different histone modifications now appear to play major roles in transcriptional regulation (Zhang, 2003; Chandrasekharan et al., 2009; Mao et al., 2014). Thus, proper chromatin modifications may allow the RNA polymerase II complexes (RNAP) to progress through open reading frames of disease resistance genes that may have been silent prior to inoculation only because they were stalled (Wu and Snyder, 2008).

The method of choice for determining if certain nuclear proteins are associated with a given PR gene was chromatin immune-precipitation (ChIP). ChIP allows the quantitation of a specific protein attached in the region of the gene at a specific time point. The live tissue is treated with formaldehyde that cross-links, in place, the gene and its attached proteins. The extracted chromatin is subsequently sonicated breaking the DNA in gene-sized fragments. Antiserum of the targeted nuclear protein is able to find and selectively separate a specific DNA-protein complex from the sonicated material. Subsequently the protein is separated in a high salt buffer from the DNA fragment and PCR probes are used to quantitate the level of the desired gene-specific DNA. The greater the PCR product–the more of the protein that was initially associated with the gene. The ChIP assay determined that both the HMG A architectural transcription factor and histones H2A, H2B were reduced in the vicinity of the DRR206 and β- glucanase genes, within 5 h after the pea tissue was inoculated with Fsph spores (Isaac et al., 2009). The reduction in the presence of these nuclear proteins on DNA throughout the pea tissue within this period was as a result of ubiquitination.

### The DNA sequence target of a PR gene inducer was identified with a DNA cross-linking psoralen

4′-aminomethy-4,5′,8-trimethylpsoralen (AMT) both activates PR genes and elicits pisatin accumulation. The AMT molecule when activated with UV light crosslinks DNA *in vivo* in regions where it was attached or intercalated. These sites of altered DNA conformation remain cross-linked through DNA extraction. The specific crosslinks were identified in the region of the PR gene, DRR 49-e, via southern analysis, following the *Hind*III digestion of pea DNA and gel separations in separate lanes. The cross-linked DNA was distinquished from single stranded fragments in basic-pH lanes verses neutral-pH lanes within the gel (Parsons and Hadwiger, 1998).

### Chromosomal changes that can be detected cytologically

Chromosomal changes that involve chromatin or the DNA damage have been pursued with different cytological approaches. An assay that measures DNA fragmentation *in situ* employed the TdT-mediated dUTP nick-end-labeling method in which the terminal deoxynucleotidyl transferase incorporates fluorescein-12-dUTP on the cleaved ends of fragmented DNA. An antifluorescein-alkaline phosphatase conjugate (TUNEL) subsequently binds dUTP-fluorescein that in the presence of Fast Red, red precipitates in those nuclei containing fragmented DNA (Klosterman et al., 2001). Enhanced fragmentation of pea DNA occurred within 5 h following treatment of pods with Fsph DNase or inoculations with Fsph or Fspi.

A portion of partially digested fungal wall components, particularly the chitosan component, have become localized in pea nuclei following inoculations (Hadwiger et al., 1981). Chitosan and other glucose containing polymers were detected intracellular initially with radiolabels and subsequently with antisera specific to Fusarium walls or to pure chitosan. Intra-cellular chitosan has been traced with both light and electron microscopic analyses (Hadwiger et al., 1981). Within 15 min after applying [^3^H]-chitosan to the endocarp surface, the label is detected within both the plant cytoplasm and nucleus. Within 5 h chitosan was also found, detected with FITC-conjugated chitosan antiserum, to be distributed around sites of fungal spore attachments. The brightest intensities seen in a fluorescence microscope were associated with the outer surface of the spore itself and less in the surrounding pea cells. Immuno-labeling specific to chitosan and *Fusarium* cell walls components were also detected in cross-sections of the host/parasite interaction prepared for electron microscope analyses. These components were found within the cell wall region and scattered within the cytoplasm of the fungus following 15 min of contact with the pea endocarp.

**Figure 2:**
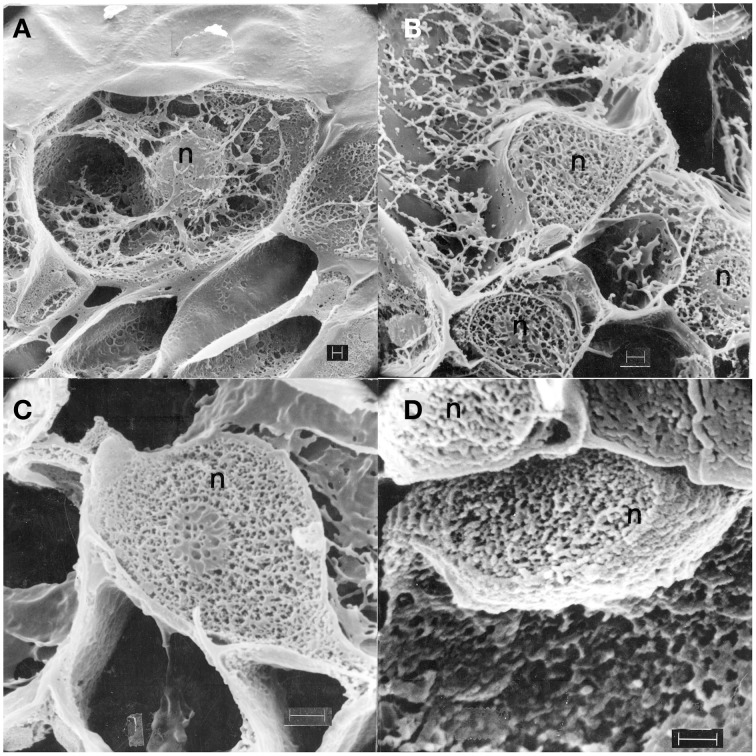
**Scanning electron micrographs (Hadwiger and Adams, 1978) showing cross-sections of freeze-fractured, freeze dried pea pod epidermal surface cells from endocarp tissue: Untreated (A), treated with water 1 h (B), inoculated with macroconidia of *Fusarium solani* f. sp. *phaseoli* 1 h (C), and 6 h (D)**. Pea nuclei, n; fungus, f; EP, Epidermis surface. Bar = 1.0 μ.

A rigorously preserved views of major changes in pea nuclear structure following inoculation with Fsph were seen in cross-sections of freeze-fractured, freeze dried preparations (Hadwiger and Adams, 1978) for the scanning electron microscope (Figure 2).

The pea nucleus from non-treated tissue is not distorted and is interconnected with cytoplasmic networks Figure 2A. Cross-sections of nuclei following 1 h of a water treatment retain a crisp structure and interconnecting cytoplasmic connections Figure 2B. Inoculations with Fsph spores commence an alteration of the nuclear netting within 1 h (Figure 2C) that becomes severe within 6 h (Figure 2D). All of the nuclei shown were in epidermal cells immediately under individual spores of the inoculum. These alterations of netting apparently reflect component alterations occurring in chromatin components. These nuclear changes are also borne out in cross-sections viewed with transmission electron microscopy (Hadwiger and Adams, 1978). As indicated above, the actual densities of nuclei in Fsph- and Fspi- challenged tissue were reduced within 20 min. The corresponding disruptions of the chromatin material within the nucleus caused within 4 h were detectable by both electron microscopy and scanning electron microscopy. The degree of DNA fragmentation has been resolved following DNA extraction and was readily detectable in CHEF gel separations (Klosterman et al., 2001).

### Functional explanations of nuclear changes

The nucleosome is a basic component of chromatin and its structure has been characterized (Luger et al., 1997) and thus is a structure encompassing a set of histones that has documented effects on gene transcription. Having established that the disease resistance response depends on an on-going ability of the plant to activate defense genes and to translate their protein products, an explanation is warranted for how transcription and translation may be influenced by the early cellular changes in chromatin. The two *Fusarium* biotic elicitors that have been defined in this interaction are chitosan and FsphDNase. Both can have a direct effect on the DNA within chromatin. Chitosan heptamers predictably can reside in the DNA minor groove causing helical changes in addition being an aggressive competitors for basic protein attachment sites on DNA (Hadwiger et al., 1989). The single strand nicking potential of FsphDNase is transferred rapidly from the spores to the pea nucleus (Gerhold et al., 1993). The close contact between spore and plant cell and the associated deterioration within the extremities of both entities is a potential source of even more eliciting components, some of which follow conventional pathways for signaling transcription by influencing the phosphorylation or other alterations of transcription factors (Weake and Workman, 2008). Recent transcription theories propose that RNA polymerase II transcription complexes (RNAP) can exist in a stalled position on DNA within promoter regions of certain genes (Wu and Snyder, 2008).

If defense genes are mainly in regions being identified by the QTL analyses of disease resistance in plants (Pilet-Nayel et al., 2002; Prioul-Gervais et al., 2007), it may explain why, that not all plant genes are influenced strictly by transcription factor changes. Further, some genes such as the Sp 2 gene in another eukaryotic system can become transcribed primarily due to dis-assembly of chromatin and yet still be dependent on transcription factors when chromatin is re-assembled (Adkins and Tyler, 2006).

The nuclear changes documented as density changes, DNA alterations, visible changes viewed by microscope or molecular analyses of fragmentation are likely destroying chromatin organization and eliciting abnormal gene activations. These abnormal changes activate defense gene transcription initially but as this level of destruction proceeds cell to cell, the benefit may turn to liability and cause cellular damage, as seen in all interactions that result in an eventual increase in cell death. In the pea endocarp system cell death occurs but does not represent a significant percentage until 9–18 h pi, well after resistance to Fsph has occurred. Alternately, since the Fsph gene is active as the *Fusarium* spore is germinating (Klosterman et al., 2001), the *inherent* levels of fungal DNA destruction must be held in check. The fungus may be required to grow rapidly enough that intact nuclei in the growing tips are not involved with the DNA destruction that normally occurs in older tissue (Griffin, 1994). This separation may be stifled when the pathogen contacts the host tissue and falls victim to the defense response that slows fungal growth. This destruction could terminate fungal growth, in a manner similar to the nuclear destruction that causes cell death in the host plant cells.

### Chromatin alterations: Evidence for direct effects on DNA

**Figure 3:**
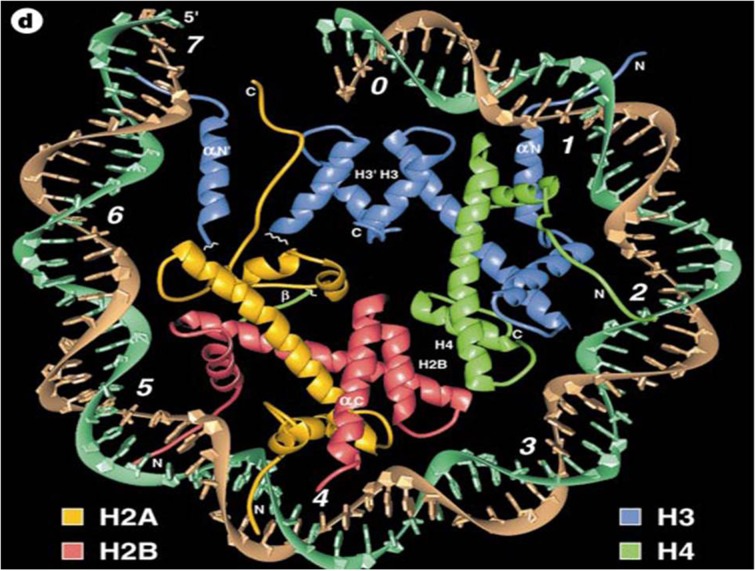
**Crystal structure of the nucleosome core particle as proposed by Luger et al. (1997) (re-published with permission from Nature 389:251)**. The DNA helical component (brown and turquoise) is portrayed on the outer part of the particle with histones, H2A, H2B, H3, and H4 within the center of the core.

As indicated in the crystal structure model of chromatin Figure 3, the basic components of nucleosomes in eukaryotic cells are highly conserved. The earliest recognition of how the alterations of chromosomes can activate of suppress gene transcription was demonstrated with the polytene or giant chromosomes of *Drosophila*. Watson et al. (1987), described it in the following way: *“Polytene chomosomes allow visualization of gene expression. The DNA decondenses in to a much more open state for a distinctive puff. The larger and more diffuse a puff appears, usually the higher the rate of its transcription. Different sets of bands can be induced by heat shock or by the addition of the insect hormone ecdyisone which stimulates the synthesis of proteins required for molting and pupation. The use of antibodies directed against various nuclear proteins can show the molecules such as RNA polymerases and isomerases become specifically concentrated in puff regions.”*

**Figure 4:**
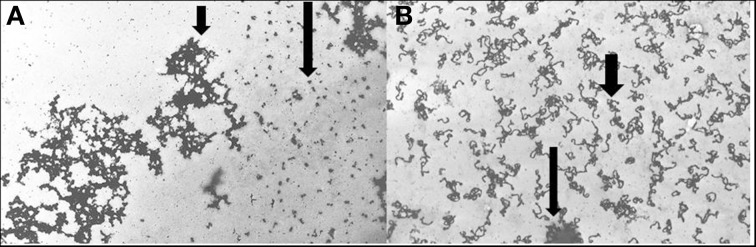
**Pea chromatin spreads. (A)** Actinomycin D-^3^H was applied 1 h to pea endocarp tissue. The silver precipitates (long arrow) represent the presence of this intercalating agent in areas of dispersed pea chromatin. The short arrow indicates condensed chromatin. **(B)** Chromatin from pea tissue treated with actinomycin D (2 μg/ml) and pulse labeled 1 h with uridine-^3^H. Larger arrow indicates an aggregate of chromatin and the broad arrow indicates the presence of RNA-^3^H. The cytological techniques are described in Hadwiger and Adams (1978).

DNA specific compounds such as actinomycin D (AD) also cause characteristic puffs in polytene chromosomes (Berendes, 1968). AD is also disruptive to pea chromatin and its specific targets can be observed in chromatin spreads as seen in Figure 4. Further, AD does not simultaneously affect all regions of pea chromatin as can be seen in the selective unraveling identified with the use of tritiated AD. When non-labeled AD is applied, at concentrations that induce phytoalexin production, along with a 1 h pulse of tritiated uridine, certain dispersed regions of the pea chromatin rapidly transcribe RNA (Figure 4B).

The RNA polymerase II complex that transcribes the open reading frame of the gene, proposedly does so by temporarily displacing the histone components that had compacted the DNA preventing the read out. For many genes in a repressed state called “stalled,” the RNA Polymerase II-containing transcription complex (RNAP) can be in place along with the appropriate transcription factors (TF) (Wu and Snyder, 2008). The presence of histones and proteins such as the HMG A (previously, HMG-I/Y) “architectural” transcription factor (TF) (Klostermann et al., 2003) can complex to DNA sequences in the vicinity of the promoters of some pathogenesis-related genes. These proteins can constitute a part of the repressed state (Zhang, 2003). HMG A has an AT-hook with specificity to AT rich regions of the DNA (Klostermann et al., 2003). Both of the biotic elicitors, chitosan heptamer and FsphDNase, have the potential to disrupt the organization of the nucleosome causing a de-repression or reversal of the stalled state. Previous reports (Choi et al., 2001; Klosterman et al., 2001) indicate that these elicitors cause DNA damage in the early hours following contact with the pea tissue.

It is likely that there additional components generated in the interaction that can augment or reduce the full defense response. Trials using chitosan-^3^H indicated 19% of this externally applied elicitor that enters the plant cell reaches the nucleus (Hadwiger et al., 1989). Likewise the FsphDNase gene product is predicted to contain an N-terminal peptide categorized as a SignalP component and its entrance into the plant nucleus has also been verified (Gerhold et al., 1993). Interestingly, the genomes of essentially all fungi sequenced to date contain an open reading frame coding a DNase gene including the predicted N-terminal SignalP component in the protein product (Hadwiger and Polashock, 2013).

## DNA damage in the fungus

**Figure 5:**
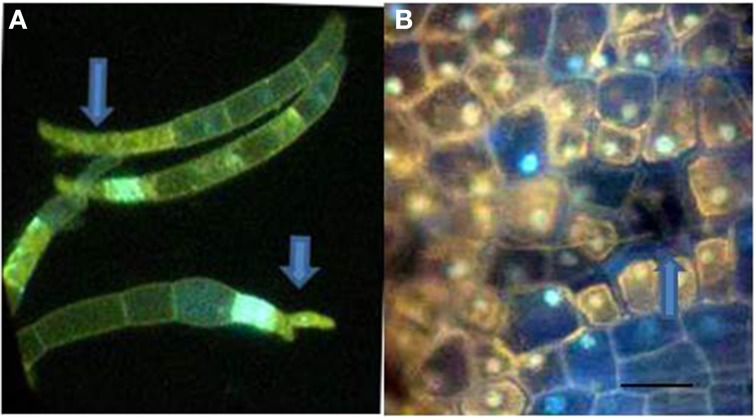
***F. solani* f.sp. *phaseoli* (Fsph) spores DAPI stained to detect fungal nuclear and plant cell DNA positive material. (A)** Fsph spores cultured in shake culture 3.5 h followed by contact with pea endocarp tissue for 2 h, viewed under UV light. Arrows point to DAPI stained tips of the spores where nuclear destruction is evident. Some spore cell compartments were devoid of detectable nuclei. **(B)** Photo of epidermal surface of a Fsph lesion encompassing both non-flourescing plant cells and a non-fluorescing, non-visible spore 20 h pi. The fluorescing nuclei in cells surrounding the spore indicate different states of the plant nuclear staining depending on their location away from the lesion. Bar = 30μ.

As reported earlier, the fungal gene for FsphDNase is active as the spore is germinating (Klosterman et al., 2001). DAPI staining of the fungus within 2 h pi revealed that the nuclei in the most advanced fungal mycelia tips undergo deterioration Figure 5. Fspi, a true pathogen of peas, also incurs DNA damage in contacting the host tissue, however the nuclei in some hyphal tips remain intact. This escape mechanism may be responsible for the Fspi return to growth and virulence at 10–18 h pi. There are numerous similarities in responses induced by Fsph and Fspi (Hadwiger, 2008), but the major molecular difference appears to reside in the speed at which the response is generated by Fsph.

## Nonhost resistance in the pea endocarp responds to some signals not implicated in other interactions

DNase genes coded with N-terminal sequences predicted to be secreted are found within the genomes of all fungi sequenced to date (Hadwiger and Polashock, 2013) and chitin or chitosan are components of many fungal walls suggesting that their signaling is universal. Alternately, a screening of elicitors capable of inducing pisatin production, indicates that the well-researched chemicals, salicylic acid (SA), jasmonic acid, flg22 and chitin oligomers are ineffective phytoalexin elicitors in pea tissue (Hartney et al., 2007). Although there is a lack of a similar immune response to SA in pea, in *Arabidopsis* SA also activates DNA damage responses to potentiate plant immunity (Yan et al., 2013). Elevated DNA damage caused by *sni1* mutation or treatment with a DNA damaging agent (Bleomycin) enhances SA-mediated expression of *Arabidopsis* defense genes PR1, and PR2. Alternately, the signaling of pea defense genes are very efficiently activated by compounds such as the phosphatase inhibitors, calyculin A, endothall, and cantharidin, indicating that cascade signaling can be ongoing without the direct DNA damage effects. Additionally, certain concentrations of these phosphatase inhibitors can also break the pea tissue's nonhost resistance against Fsph (Hartney et al., 2007).

## Conclusions based on this condensed pea/Fsph reaction time for disease resistance, can discount implications of events observed significantly later in systems researching longer terms pi

The pea endocarp/*Fusarium solani* sp. system has unique advantages for examining the major players in nonhost disease resistance in the first 6 h pi, due to an absence of an interfering cuticle layer. Also, as indicated previously, simultaneous spore to plant cell signaling involves the total epidermal layer, enabling the simultaneous and synchronized progression of molecular events within a high percentage of the cells in this intact tissue. Further, the artifacts inherent to leaf disk and cell culture assays are by-passed. Taken together all the data discussed above, a case can be made that the transcription of the defense response genes can be initiated directly at the intra-cellular point of transcription, the chromatin. The speed of this initiation is important, since as reported earlier (Hadwiger, 2008), the biochemical characteristics of the resistance and susceptible responses vary mainly in the rate at which the PR genes and other responses are developed. Within the slower susceptible response, the true pathogen is able to maintain a small number of its nuclei intact, allowing it to resume growth after the major surge of the resistance response has peaked out (Klosterman et al., 2001).

## Programed cell death and reactive oxygen species (ROS)

Since there is a cryptic window in which disease resistance develops in the pea endocarp system, can it divert the focus from previously reported explanations of resistance via programmed cell death? The plant cell death has been considered in other systems, as a means for the plant to localize the pathogen to a restricted lesion within a few plant cells. The appearance of cytologically-significant or dye-detectable pea cell death does occur but not before 6 h pi (Choi et al., 2001). At 9 h pi there was an elevation to 3% cell death in the pea endocarp tissue challenge by Fsph and up to 10% in the challenge by Fspi. The Fspi challenge is accompanied with aggressive Fspi growth at 18 h, suggesting that in this system the delayed cell death favored susceptibility. In some systems increases in reactive oxygen species (ROS) reportedly serve as a signal to activate the defense response (Scheel, 1998; Rojas et al., 2012). The effect of the release of ROS, or the scavengering of ROS on the pea endocarp tissue does not demonstrate-ably alter nonhost defense responses following the application of compounds that reportedly release or scavenger ROS (Hartney et al., 2007). Pea endocarp applications of chemicals such as methotrexate that produces reactive oxygen species; N-acetyl-cysteine that increases free radical scavengers, citrulline and L-arginine substrates for NO synthesis reportedly do not interfere with the major signaling in pea/Fusarium interactions (Hartney et al., 2007). ROSs reportedly can incite DNA damage through multiple base lesions (Cooke et al., 2003). Because of the transient properties of ROS and their potential to damage DNA, they can't currently be included or excluded as potential gene activators in peas.

## Conclusion

The pea/*Fusarium* interactions within the critical 6 h window in which nonhost resistance develops, represent a form of cellular incompatibility resulting from component exchange between the fungal spore and plant cell. Nonhost resistance is initiated by multiple signals that can differ biochemically as extensively, as the elicitors: chitosan heptamer, Fsph DNase, and actinomycin D. Even with this diversity the resulting induced protein patterns and including the plant PR proteins transcribed are surprisingly similar. In pea endocarp tissue all of these signals appear to target DNA. Fungal wall components such as chitosan and the fungal DNase gene product, are released and nuclear localized. These authentic biotic elicitors are ubiquitous among fungi. I propose that they are the major signals of nonhost resistance in peas and act by unblocking stalled RNA polymerase II complexes poised at PR gene promoter sites. The affected genes are likely those located within the sensitive regions (e.g., QTLs) of pea chromosomes. The mechanism by which the fungal growth is suppressed is initially from the antifungal properties of chitosan oligomers, plant defensins and other PR gene products. This growth suppression rapidly contributes to the inherent accumulation of FsphDNase and other hydrolytic enzymes of fungal origin within the fungus itself. The resultant destruction of hyphal tip nuclei definitively terminates fungal growth.

### Conflict of interest statement

The author declares that the research was conducted in the absence of any commercial or financial relationships that could be construed as a potential conflict of interest.
